# A systematic review and meta-analysis on mortality rate following total pelvic exenteration in cancer patients

**DOI:** 10.1186/s12885-024-12377-5

**Published:** 2024-05-15

**Authors:** Arezoo Esmailzadeh, Mohammad Sadegh Fakhari, Nafise Saedi, Nasim Shokouhi, Amir Almasi-Hashiani

**Affiliations:** 1 Department of Obstetrics & Gynecology, Trauma Research Center, Baqiyatallah University of Medical Sciences, Tehran, Iran; 2 Student Research Committee, Arak University of Medical Sciences, Arak, Iran; 3 Fellowship of Perinatology, Department of Gynecologic Oncology, Tehran University of Medical Sciences, Tehran, Iran; 4 Fellowship of Female Pelvic Medicine and Reconstructive Surgery, Yas Women Hospital, Tehran University of Medical Sciences, Tehran, Iran; 5 Department of Epidemiology, Arak University of Medical Sciences, Arak, Iran; 6 Traditional and Complementary Medicine Research Center, Arak University of Medical Sciences, Arak, Iran

**Keywords:** Total pelvic exenteration, Mortality, Colorectal neoplasms, Gynecological neoplasms, Urologic neoplasms

## Abstract

**Background:**

Total pelvic exenteration (TPE), an en bloc resection is an ultraradical operation for malignancies, and refers to the removal of organs inside the pelvis, including female reproductive organs, lower urological organs and involved parts of the digestive system. The aim of this meta-analysis is to estimate the intra-operative mortality, in-hospital mortality, 30- and 90-day mortality rate and overall mortality rate (MR) following TPE in colorectal, gynecological, urological, and miscellaneous cancers.

**Methods:**

This is a systematic review and meta-analysis in which three international databases including Medline through PubMed, Scopus and Web of Science on November 2023 were searched. To screen and select relevant studies, retrieved articles were entered into Endnote software. The required information was extracted from the full text of the retrieved articles by the authors. Effect measures in this study was the intra-operative, in-hospital, and 90-day and overall MR following TPE. All analyzes are performed using Stata software version 16 (Stata Corp, College Station, TX).

**Results:**

In this systematic review, 1751 primary studies retrieved, of which 98 articles (5343 cases) entered into this systematic review. The overall mortality rate was 30.57% in colorectal cancers, 25.5% in gynecological cancers and 12.42% in Miscellaneous. The highest rate of mortality is related to the overall mortality rate of colorectal cancers. The MR in open surgeries was higher than in minimally invasive surgeries, and also in primary advanced cancers, it was higher than in recurrent cancers.

**Conclusion:**

In conclusion, it can be said that performing TPE in a specialized surgical center with careful patient eligibility evaluation is a viable option for advanced malignancies of the pelvic organs.

## Introduction

Total pelvic exenteration (TPE), an *en bloc* resection is an ultraradical operation for malignancies which was performed for the first time in 1946 by Alexander Brunschwig [1], and refers to the removal of organs inside the pelvis, including female reproductive organs, lower urological organs and involved parts of the digestive system (rectosigmoid) [2–4].

TPE procedure is used in the treatment of advanced gynecological cancers as well as primary advanced and recurrent rectal cancers [3, 5]. Even though TPE is infrequently performed, it may be considered as the last hope for the treatment of recurrent or advanced cancers [6, 7].

TPE technique was associated with significant complications and mortality in the first decades, but in recent decades due to the improvement of preoperative planning (whole-body positron emission tomography), intraoperative and postoperative care, the survival rate, surgical complications and mortality of candidate patients has improved significantly [4, 8, 9].

Overall survival and disease-free survival rate significantly improved following TPE, especially in well-selected patients [3]. To the best of our knowledge, the highest 5 years overall survival rate was reported as 65.8% [10] in cervical cancer patients following pelvic exenteration and in colorectal cancer patients, one year survival rate was more than 80% in several previous studies [11–14] and the highest five year survival rate was reported as 92.9% in a study by Mark Katory et al. in the United Kingdom [14].

Considering that this surgical technique is considered a rare and advanced technique, significant complications and mortality rate (MR) have been reported for it. Intra-operative mortality, in-hospital mortality, 30- and 90-day mortality are important consequences that are reported for the management of the complications of this surgery. In addition to the survival rate, mortality and complications are also changing over time and depend on the equipment of the surgical center as well as the experience of the surgical team, and different studies have reported different mortality rates and there is no comprehensive review in this regard. The aim of this meta-analysis is to estimate the intra-operative mortality, in-hospital mortality, 30- and 90-day mortality rate and overall mortality rate following TPE in colorectal, gynecological, urological, and miscellaneous.

## Methods

### Study design

This is a systematic review and meta-analysis in which international databases were searched to find the relevant studies. Standard guideline of “Preferred Reporting Items for Systematic Reviews and Meta-Analyses (PRISMA) was followed to prepared the report. This study was registered in the PROSPERO (CRD42023467479).

### Eligibility criteria

**Fig. 1:**
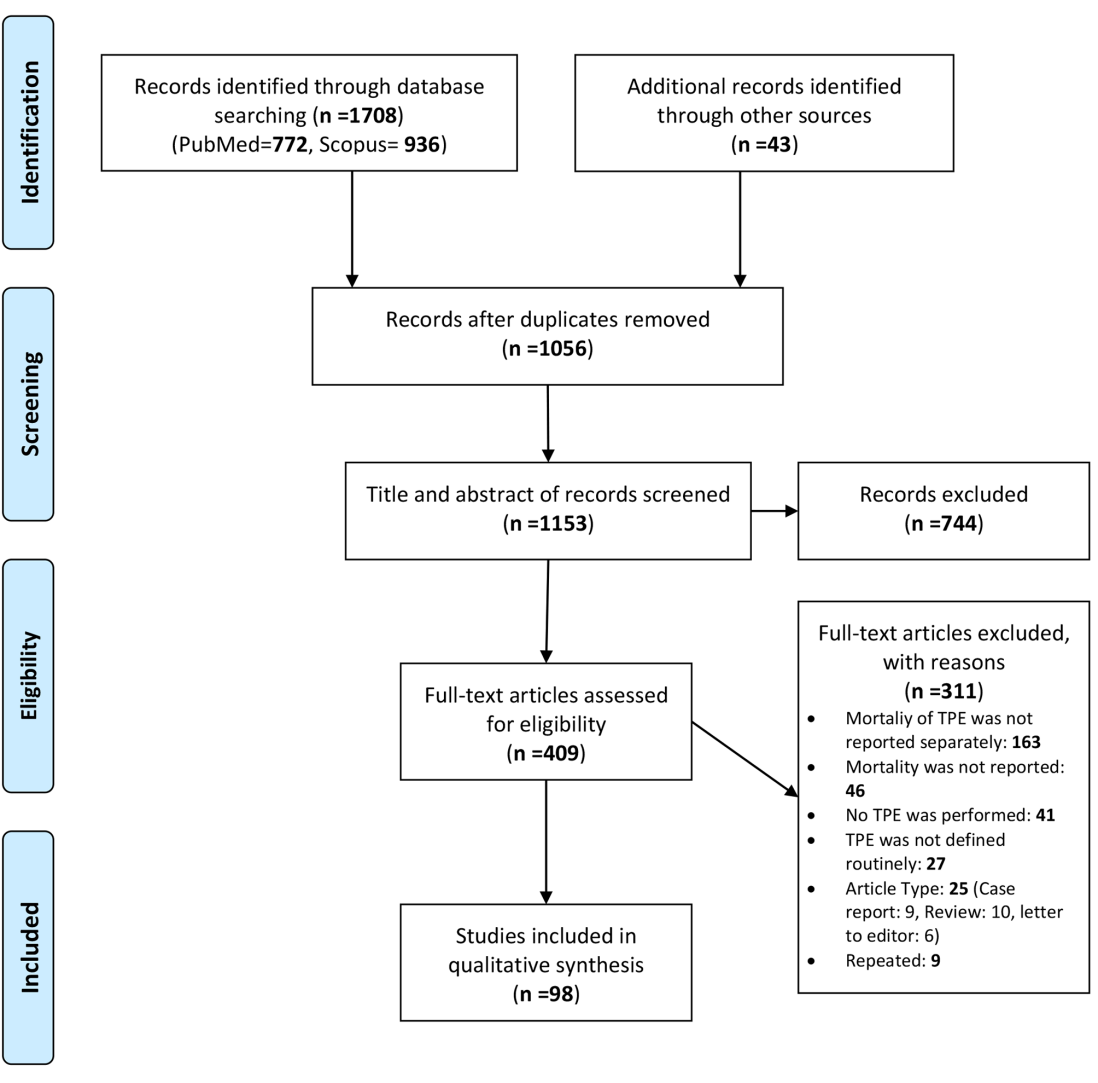
Flow diagram of the literature search for studies included in meta-analysis. TPE: Total Pelvic Exenteration

In this study, all observational studies related to the MR after TPE surgery with English full-text were included in the study. There was no time limit for entering the articles, and also in terms of the study design, all the articles that reported the MR including cohort studies, cross-sectional studies and case series studies were included. However, studies which had not defined the surgical procedure of TPE routinely were excluded. Additionally, we excluded case reports, letters to the editor, and review studies from our analysis. Although, we thoroughly screened the full texts of these articles to ensure that any relevant studies that were initially overlooked, were included in our primary search. Further details of the excluded articles are defined in Fig. 1.

### Information sources and search strategy

Articles published in English were searched. To retrieved relevant articles, the search was carried out using keywords for three international databases including Medline through PubMed, Scopus and Web of Science on November 2023. Different keywords were used to search the databases, and the search strategy in PubMed is given as an example.

((((“Survival“[Mesh] OR “Mortality“[Mesh] OR “mortality” [Subheading] OR “Disease-Free Survival“[Mesh] OR “Survival Analysis“[Mesh] OR “Survival Rate“[Mesh]) OR (“Survival“[tw])) OR (“Mortality“[tw])) OR (“Disease-Free Survival“[tw])) AND (((((“Pelvic Exenteration“[Mesh]) OR (“Pelvic Exenteration“[tw])) OR (“Pelvic Exenteration“[tiab])) OR (“total Pelvic Exenteration“[tw])) OR (“total Pelvic Exenteration“[tiab]))

### Data collection process

To select relevant studies, retrieved articles were entered into Endnote software and duplicate articles were removed at this stage. Then the titles and abstracts of the remaining articles were screened and irrelevant articles were discarded. After that, the full text of the remaining articles was evaluated and irrelevant articles were removed. Finally, the required information was extracted from the remaining related articles.

### Data items

The required information was extracted from the full text of the retrieved articles by the authors, and in cases of disagreement, decisions were made in consultation with other authors. The data extracted from each article included the name of the first author, year of publication, type of study design, sample size, type of cancer, location of the study, MR, included sample size, quality score of studies and the study population.

Our results were divided into four groups based on type of cancer: colorectal, gynecological, urological, and miscellaneous. The miscellaneous category included data on MR of TPE regardless of cancer type. Other cancers indicated for TPE in this study included squamous cell carcinoma, soft tissue sarcoma, perineal skin cancer, anal cancer, leiomyosarcoma, etc.

MR for intra-operative mortality, in-hospital mortality, 30-and 90-day mortality, was defined as reported deaths due to the surgical procedure and MR for overall mortality included death of the patients during the follow-up period due to surgery or cancer. Notably, patients who died due to other causes or were lost to follow-up were omitted from the analysis.

### Study risk of bias assessment

The Joanna Briggs Institute Critical Appraisal (JBI) checklists were used to assess the quality of the included studies [15]. For each type of article, either cohort studies or case series, we utilized the relevant checklists provided by JBI. Each item on the checklist was assigned a score of 1 if the response was “yes”, and 0 if the response was “no”, “unclear”, or “not applicable”. The quality indicators were converted to 100%, Studies addressing ≥ 75% of the checklist items were considered as having a low risk of bias [16]. One author (MSF) carried out the quality assessment.

### Effect measures

Effect measures in this study was the intra-operative, in-hospital, 30-day and 90-day and overall MR following TPE. The included sample size and the number of dead people were extracted from the studies, and the MR and 95% confidence interval were calculated.

### Synthesis methods and statistical analysis

To check the heterogeneity among the studies, the I^2^ statistic was used and it was tested using the chi-square test, and if there was significant heterogeneity between the studies, the random-effects model was used to merge the data. Although, based on the heterogeneity between the studies, from a methodological point of view, the fixed effects model was used, but considering that the mortality rate may be different based on the center expertise, surgeon experience, and postoperative care, in addition to the fixed effects model, random effects model was also performed and its results were reported. Egger’s linear regression test, Begg’s test and funnel plot were used to check publication bias. All analyzes are performed using Stata software version 16 (Stata Corp, College Station, TX).

## Results

### Study selection

In this systematic review, 1751 primary studies (772 papers via Medline, 936 via Scopus and 43 papers via additional search) retrieved. Of the total articles, 695 duplicate articles were identified and removed. Then, the titles and abstracts of the remaining 1153 articles were screened and at this stage, 744 articles were excluded due to the lack of fulfilling the inclusion criteria and the full text of 409 remained articles was evaluated, of which 98 articles entered into this systematic review. All the process was presented in Fig. 1.

### Study characteristics

As it was shown in Tables 1 and 98 studies [6, 7, 12–14, 17–102] (5343 cases) were included in the analysis. The oldest one was published in 1967 and the most recent in 2023. Both case series (23 studies) and cohort studies (75 studies) were included in the analysis. The sample size of included studies ranged from 2 to 2305 cases and colorectal, gynecological, urological and miscellaneous cancers were included in the analysis. More details in this regard were presented in Table 1.

**Table 1:** Characteristics of Included Studies

Author	Publish Year	Country	Case Series	Cancer Type	Cancer Origin	Surgery Method	Sample Size	Median Follow up time (month)	Inrta-operative MR	In-hospital MR	30-day MR	90-day MR	Overall MR
Ingiulla et al. [17]	1967	Italy	Cohort	Gynecological	Both	Open	51	NR			26		
Thornton et al. [18]	1973	USA	Case Series	Gynecological	Primary	Open	7	18.5 (range: 7-114)		1	1		3
Karlen et al. [19]	1975	USA	Cohort	Gynecological	Both	Open	29	NR		7			16
Eckhauser et al. [20]	1979	USA	Case Series	Colorectal	Primary	Open	10	NR	1	1	1	1	1
Ledesma et al. [21]	1981	USA	Cohort	Colorectal	Both	Open	30	NR		3			
Mori et al. [22]	1981	Japan	Cohort	Misc.	Both	Open	21	NR	1	1	3		
Boey et al. [23]	1982	China	Cohort	Colorectal	Both	Open	26	NR			4	7	
Takagi et al. [24]	1985	Japan	Case Series	Colorectal	Primary	Open	13	25 (range: 1-132)	0	0	1		7
Cuevas et al. [25]	1988	USA	Cohort	Gynecological	Both	Open	120	36			27		
Yeung et al. [26]	1993	Canada	Cohort	Colorectal	Both	Open	50	NR		7	4		
Liu et al. [27]	1994	China	Cohort	Colorectal	Both	Open	31	NR	0	0	0	0	16
Lopez et al. [28]	1994	USA	Cohort	Misc.	Both	Open	232	NR					34
Sardi et al. [29]	1994	USA	Case Series	Colorectal	Recurrent	Open	6	NR	0	0	0	0	1
Woodhouse et al. [30]	1995	UK	Case Series	Colorectal	Primary	Open	2	NR		0			
				Gynecological			3			0			
				Misc.			6			0			
				Urological			1			0			
Luna-Perez et al. [31]	1996	USA	Cohort	Colorectal	Primary	Open	12	46 (range:3-148)			2		
Shirouzu et al. [32]	1996	Japan	Case Series	Colorectal	Both	Open	26	NR			2		
Russo et al. [12]	1999	USA	Cohort	Colorectal	Both	Open	47	16.83			1		20
Law et al. [33]	2000	China	Cohort	Colorectal	Both	Open	24	mean:49.8 (range:6-160)	0	0	0	0	8
Chen et al. [34]	2001	Taiwan	Cohort	Colorectal	Primary	Open	50	NR	0	1	1	1	4
Wiig et al. [35]	2002	Norway	Cohort	Colorectal	Primary	Open	47	60	0	5	2		
Ike et al. [36]	2003	Japan	Cohort	Colorectal	Primary	Open	71	NR	1	3			25
Jimenez et al. [37]	2003	USA	Cohort	Colorectal	Both	Open	55	26 (range:0.26–106)	0	0	0	3	20
					Recurrent		39		0	0	0		
					Primary		16		0	0	0		
Kamat et al. [13]	2003	USA	Case Series	Urological	Recurrent	Open	14	14 (range: 3–36)	0	0	0	0	7
Vitelli et al. [39]	2003	Italy	Cohort	Colorectal	Both	Open	8	40 (range:12–120)			2		
Houvenaeghel et al. [40]	2004	France	Cohort	Gynecological	Both	Open	55	NR			5		
Berek et al. [6]	2005	USA	Cohort	Gynecological	Recurrent	Open	46	50	0				
Leibovici et al. [42]	2005	USA	Case Series	Urological	Recurrent	Open	5	range: 5–55	0	0	0		
Nguyen et al. [43]	2005	UK	Cohort	Colorectal	Both	Open	16	12.5 (range:1-120)	0	0	0		
				Gynecological			16		0	0	0		
				Misc.			41		0	0	0		
Goldberg et al. [7]	2006	USA	Cohort	Gynecological	Recurrent	Open	103	NR	0	1	1	2	2
Ferron et al. [107]	2006	France	Case Series	Gynecological	Primary	MIPE	1	total 16	0	0	0	0	0
de Wilt et al. [44]	2007	Netherlands	Cohort	Gynecological	Both	Open	17	42 (range:1-155)	0	0	0		
Park et al. [45]	2007	South Korea	Cohort	Gynecological	Both	Open	30	NR	0	0	0		
Ungar et al. [48]	2008	Hungary	Cohort	Gynecological	Primary	Open	2	NR		0	0		
Vermaas et al. [46]	2008	Netherlands	Cohort	Colorectal	Both	Open	35	mean: 28		1			
Ferenschild et al. [49]	2009	Netherlands	Cohort	Misc.	Both	Open	69	43 (range:1-196)		1			
Maggioni et al. [50]	2009	Italy	Cohort	Gynecological	Both	Open	48	22.3 (range:1.6–117)	0	0	0		
Puntambekar et al. [51]	2009	India	Case Series	Gynecological	Primary	MIPE	7	11 (range: 4–24)	0	0	0	0	4
Spahn et al. [52]	2010	Switzerland	Cohort	Gynecological	Both	Open	6	30.5 (range:2-144)	0	0	0		
Zoucas et al. [53]	2010	Sweden	Cohort	Misc.	Both	Open	32	NR	0	0	0	0	
Chokshi et al. [54]	2011	USA	Cohort	Colorectal	Both	Open	36	NR	0	0	0		
				Gynecological			6		0	0	0		
				Misc.			53		0	0	0		
				Urological			5		0	0	0		
Domes et al. [55]	2011	Canada	Cohort	Colorectal	Both	Open	28	35 (range: 1-147)		1	1		9
Guimarães et al. [56]	2011	Brazil	Case Series	Gynecological	Recurrent	Open	13	mean: 8	2	2	2		10
Mitulescu et al. [57]	2011	Romania	Cohort	Colorectal	Both	Open	48	NR	0				
				Gynecological			159		0				
				Misc.			213		0				
				Urological			4		0				
Baiocchi et al. [58]	2012	Brazil	Cohort	Gynecological	Both	Open	56	13.8 (range: 1.09–114.3)	0				
Kuhrt et al. [59]	2012	USA	Cohort	Colorectal	Both	Open	36	NR	0	0	0		
				Gynecological			6		0	0	0		
				Misc.			53		0	0	0		
				Urological			3		0	0	0		
Ramamurthy et al. [60]	2012	India	Cohort	Colorectal	Primary	Open	3	36 (range: 11–76)	0	0	0		
				Gynecological			10		0	0	0		
				Misc.			13		0	0	0		
Yoo et al. [61]	2012	South Korea	Cohort	Gynecological	Recurrent	Open	42	22 (range: 1–60)	0	0	0		
Jäger et al. [62]	2013	Sweden	Cohort	Gynecological	Recurrent	Open	11	27 (range: 2-110)	0	0	0		
Tan et al. [63]	2013	Australia	Cohort	Misc.	Recurrent	Open	10	26 (range: 4–169)	0	0	0		
Ueda et al. [64]	2013	Japan	Cohort	Misc.	Both	Open	13	25.5	0	0	0		
Ghouti et al. [71]	2014	France	Cohort	Colorectal	Recurrent	Open	14	33.5 (95%CI: 25.4–36.9)	0	0	0		
milne et al. [65]	2014	Australia	Cohort	Misc.	Both	Open	68	NR	0	0	0		
Pathiraja et al. [66]	2014	UK	Cohort	Gynecological	Both	Open	9	NR	0	0	0		
Petruzziello et al. [67]	2014	Brazil	Cohort	Gynecological	Both	Open	14	NR	0	3			
Tanaka et al. [68]	2014	Japan	Case Series	Gynecological	Recurrent	Open	3	22 (range:3-116)	0	0	0		2
Xin et al. [69]	2014	Singapore	Case Series	Colorectal	Both	Open	5	23	0	0	0		
Căpîlna et al. [70]	2015	Romania	Case Series	Gynecological	Both	Open	6	NR	0				
Kusters et al. [72]	2015	Netherlands	Cohort	Colorectal	Both	Open	23	62 (range: 2-191)	0	0	0		
Moreno-Palacios et al. [73]	2015	Spain	Case Series	Gynecological	Recurrent	Open	8	14 (range: 5–69)	0	0	0	0	3
Rombouts et al. [74]	2015	Australia	Cohort	Colorectal	Recurrent	Open	48	NR	0	0	0		
Ogura et al. [108]	2015	Japan	Cohort	Colorectal	Both	Open	15	NR	0	0	0		
						MIPE	9		0	0	0		
Yang et al. [109]	2015	China	Case Series	Misc.	Both	MIPE	11	mean: 11.1 (range: 2–24)	0	0	0	0	2
Schmidt et al. [76]	2016	Switzerland	Cohort	Gynecological	Both	Open	34	35 (range: 1-263)	0				
Chew et al. [77]	2017	Singapore	Cohort	Misc.	Both	Open	10	17.6	0	0	0		
Katory et al. [14]	2017	UK	Cohort	Colorectal	Both	Open	14	30.4 (range: 0.1–83.8)			3		
Aslim et al. [78]	2018	Singapore	Cohort	Urological	Both	Open	10	11.7 (range: 4.2–47.6)	0	0	0		
Hagemans et al. [79]	2018	Netherlands	Cohort	Colorectal	Both	Open	126	NR		6	7	11	21
Li et al. [80]	2018	China	Cohort	Gynecological	Both	Open	20	28 (range: 12–96)	0	0	0	2	
Mehta et al. [81]	2018	UK	Case Series	Colorectal	Both	Open	10	15 (IQR:8–37)	0	0	0	0	
Rema et al. [89]	2018	India	Cohort	Gynecological	Recurrent	Open	17	27.5 (Range: 1.8–99.1)	0				
Romeo et al. [82]	2018	Argentina	Cohort	Gynecological	Both	Open	15	20.3 (range: 1–60)	0				
Tortorella et al. [91]	2018	USA	Cohort	Gynecological	Both	Open	45	NR	0	0	0		
Pokharkar et al. [110]	2018	India	Case Series	Colorectal	Primary	MIPE	10	NR	0	0			
Bizzarri et al. [83]	2019	Italy	Cohort	Gynecological	Both	MIPE	5	15 (range: 6–37)	0	0	0		
Gregorio et al. [84]	2019	Germany	Cohort	Gynecological	Both	Open	10		0	0	0		
Kiiski et al. [85]	2019	Finland	Cohort	Gynecological	Both	Open	26	35.1 (range 2.5–123)	0	0	0		
Lago et al. [86]	2019	Spain	Cohort	Gynecological	Both	Open	15	18. 5 (range 1–71)	0	0	0		
Lee et al. [87]	2019	Australia	Case Series	Colorectal	Both	Open	7	7 (range: 2–10)	0	0	0		
				Gynecological			3		0	0	0		
				Misc.			10		0	0	0		
Nedyalkov et al. [88]	2019	Bulgaria	Cohort	Gynecological	Both	Open	9	52.3 (range, 2.3–99.3)					7
Soeda et al. [90]	2019	Japan	Case Series	Gynecological	Both	Open	7	27.5 (median: 12)	0				2
Ichihara et al. [111]	2019	Japan	Cohort	Colorectal	Both	MIPE	10	NR	0	0	0	0	
						Open	7		0	0	0	0	
Lewandowska et al. [92]	2020	Poland	Cohort	Gynecological	Primary	Open	22	NR	0	1	0		
					Recurrent	Open	2				0		
Tuech et al. [93]	2020	France	Case Series	Colorectal	Both	Open	16	NR			2		8
Vigneswaran et al. [94]	2020	USA	Cohort	Colorectal	Both	Open	749	NR			11		
				Gynecological			335				4		
				Misc.			2305				41		
				Urological			1025				22		
Nonaka et al. [112]	2020	Japan	Cohort	Colorectal	Both	MIPE	4	NR	0	0	0		
Bogner et al. [95]	2021	Germany	Cohort	Colorectal	Both	Open	37	19.4 (IQR 10.0–32.9)	0		0		
				Gynecological			14		0		0		
				Misc.			63		0	2	0	1	2
Brown et al. [96]	2021	Australia	Case Series	Colorectal	Recurrent	Open	2	11.5 (Range: 2–18)	0	0	0	0	
Kanao et al. [97]	2021	Japan	Cohort	Gynecological	Recurrent	MIPE	7	23.1 (Range: 8.7–39.0)	0	0	0	0	0
Nielsen et al. [102]	2022	Denmark	Cohort	Misc.	Both	Open	195	23 (range: 0.5–72)			1	6	
Rios-Doria et al. [113]	2022	USA	Cohort	Gynecological	Both	Open	62	27.6 (range, 1.0–117.5)	0		0		
Karkia et al. [114]	2022	UK	Case Series	Gynecological	Recurrent	MIPE	1	total 60	0	0	0	0	0
Abdulrahman et al. [99]	2022	UK	Cohort	Gynecological	Both	Open	5	69 (range: 2–206)	0	0	0		
Quyn et al. [115]	2023	UK	Cohort	Colorectal	Both	Open	13	19.5 (IQR 7.9– 53.5 )	0	0	0	0	
Naha et al. [116]	2023	USA	Cohort	Misc.	Both	Open	792	NR			14		
Ralston et al. [117]	2023	UK	Cohort	Colorectal	Both	Open	120	36					37
Saqib et al. [118]	2023	UK	Cohort	Misc.	Both	MIPE	3	21 (range: 3–53)	0	0	0		
Beppu et al. [119]	2023	Japan	Cohort	Misc.	Both	MIPE	24	22 (range: 2–45)	0	0	0		
Valstad et al. [120]	2023	Norway	Cohort	Gynecological	Both	Open	8	59.28	0	0	0		

### Risk of bias within studies

All the articles we reviewed met over 80% of the criteria in the JBI checklists and were thus included in the study. Tables 2 and 3 describe the details of evaluating the included studies according to JBI checklist for cohort studies and case series, respectively.

**Table 2:** Quality assessment of cohort studies according to JBI checklist

Author	Publish Year	Country	Q1	Q2	Q3	Q4	Q5	Q6	Q7	Q8	Q9	Q10	Q11	Quality Score (%)
Ingiulla et al. [17]	1967	Italy	NA	NA	Y	Y	Y	Y	Y	Y	Y	Y	Y	81.82
Karlen et al. [19]	1975	USA	NA	NA	Y	Y	Y	Y	Y	Y	Y	Y	Y	81.82
Ledesma et al. [21]	1981	USA	NA	NA	Y	Y	Y	Y	Y	Y	Y	Y	Y	81.82
Mori et al. [22]	1981	Japan	NA	NA	Y	Y	Y	Y	Y	Y	Y	Y	Y	81.82
Boey et al. [23]	1982	China	NA	NA	Y	Y	Y	Y	Y	Y	Y	Y	Y	81.82
Cuevas et al. [25]	1988	USA	NA	NA	Y	Y	Y	Y	Y	Y	Y	Y	Y	81.82
Yeung et al. [26]	1993	Canada	NA	NA	Y	Y	Y	Y	Y	Y	Y	Y	Y	81.82
Liu et al. [27]	1994	China	NA	NA	Y	Y	Y	Y	Y	Y	Y	Y	Y	81.82
Lopez et al. [28]	1994	USA	NA	NA	Y	Y	Y	Y	Y	Y	Y	Y	Y	81.82
Luna-Perez et al. [31]	1996	USA	NA	NA	Y	Y	Y	Y	Y	Y	Y	Y	Y	81.82
Russo et al. [12]	1999	USA	NA	NA	Y	Y	Y	Y	Y	Y	Y	Y	Y	81.82
Law et al. [33]	2000	China	NA	NA	Y	Y	Y	Y	Y	Y	Y	Y	Y	81.82
Chen et al. [34]	2001	Taiwan	NA	NA	Y	Y	Y	Y	Y	Y	Y	Y	Y	81.82
Wiig et al. [35]	2002	Norway	NA	NA	Y	Y	Y	Y	Y	Y	Y	Y	Y	81.82
Ike et al. [36]	2003	Japan	NA	NA	Y	Y	Y	Y	Y	Y	Y	Y	Y	81.82
Jimenez et al. [37]	2003	USA	NA	NA	Y	Y	Y	Y	Y	Y	Y	Y	Y	81.82
Vitelli et al. [39]	2003	Italy	NA	NA	Y	Y	Y	Y	Y	Y	Y	Y	Y	81.82
Houvenaeghel et al. [40]	2004	France	NA	NA	Y	Y	Y	Y	Y	Y	Y	Y	Y	81.82
Berek et al. [6]	2005	USA	NA	NA	Y	Y	Y	Y	Y	Y	Y	Y	Y	81.82
Nguyen et al. [43]	2005	UK	NA	NA	Y	Y	Y	Y	Y	Y	Y	Y	Y	81.82
Goldberg et al. [7]	2006	USA	NA	NA	Y	Y	Y	Y	Y	Y	Y	Y	Y	81.82
de Wilt et al. [44]	2007	Netherlands	NA	NA	Y	Y	Y	Y	Y	Y	Y	Y	Y	81.82
Park et al. [45]	2007	South Korea	NA	NA	Y	Y	Y	Y	Y	Y	Y	Y	Y	81.82
Ungar et al. [48]	2008	Hungary	NA	NA	Y	Y	Y	Y	Y	Y	Y	Y	Y	81.82
Vermaas et al. [46]	2008	Netherlands	NA	NA	Y	Y	Y	Y	Y	Y	Y	Y	Y	81.82
Ferenschild et al. [49]	2009	Netherlands	NA	NA	Y	Y	Y	Y	Y	Y	Y	Y	Y	81.82
Maggioni et al. [50]	2009	Italy	NA	NA	Y	Y	Y	Y	Y	Y	Y	Y	Y	81.82
Spahn et al. [52]	2010	Switzerland	NA	NA	Y	Y	Y	Y	Y	Y	Y	Y	Y	81.82
Zoucas et al. [53]	2010	Sweden	NA	NA	Y	Y	Y	Y	Y	Y	Y	Y	Y	81.82
Chokshi et al. [54]	2011	USA	NA	NA	Y	Y	Y	Y	Y	Y	Y	Y	Y	81.82
Domes et al. [55]	2011	Canada	NA	NA	Y	Y	Y	Y	Y	Y	Y	Y	Y	81.82
Mitulescu et al. [57]	2011	Romania	NA	NA	Y	Y	Y	Y	Y	Y	Y	Y	Y	81.82
Baiocchi et al. [58]	2012	Brazil	NA	NA	Y	Y	Y	Y	Y	Y	Y	Y	Y	81.82
Kuhrt et al. [59]	2012	USA	NA	NA	Y	Y	Y	Y	Y	Y	Y	Y	Y	81.82
Ramamurthy et al. [60]	2012	India	NA	NA	Y	Y	Y	Y	Y	Y	Y	Y	Y	81.82
Yoo et al. [61]	2012	South Korea	NA	NA	Y	Y	Y	Y	Y	Y	Y	Y	Y	81.82
Jäger et al. [62]	2013	Sweden	NA	NA	Y	Y	Y	Y	Y	Y	Y	Y	Y	81.82
Tan et al. [63]	2013	Australia	NA	NA	Y	Y	Y	Y	Y	Y	Y	Y	Y	81.82
Ueda et al. [64]	2013	Japan	NA	NA	Y	Y	Y	Y	Y	Y	Y	Y	Y	81.82
Ghouti et al. [71]	2014	France	NA	NA	Y	Y	Y	Y	Y	Y	Y	Y	Y	81.82
milne et al. [65]	2014	Australia	NA	NA	Y	Y	Y	Y	Y	Y	Y	Y	Y	81.82
Pathiraja et al. [66]	2014	UK	NA	NA	Y	Y	Y	Y	Y	Y	Y	Y	Y	81.82
Petruzziello et al. [67]	2014	Brazil	NA	NA	Y	Y	Y	Y	Y	Y	Y	Y	Y	81.82
Kusters et al. [72]	2015	Netherlands	NA	NA	Y	Y	Y	Y	Y	Y	Y	Y	Y	81.82
Rombouts et al. [74]	2015	Australia	NA	NA	Y	Y	Y	Y	Y	Y	Y	Y	Y	81.82
Ogura et al. [108]	2015	Japan	NA	NA	Y	Y	Y	Y	Y	Y	Y	Y	Y	81.82
Schmidt et al. [76]	2016	Switzerland	NA	NA	Y	Y	Y	Y	Y	Y	Y	Y	Y	81.82
Chew et al. [77]	2017	Singapore	NA	NA	Y	Y	Y	Y	Y	Y	Y	Y	Y	81.82
Katory et al. [14]	2017	UK	NA	NA	Y	Y	Y	Y	Y	Y	Y	Y	Y	81.82
Aslim et al. [78]	2018	Singapore	NA	NA	Y	Y	Y	Y	Y	Y	Y	Y	Y	81.82
Hagemans et al. [79]	2018	Netherlands	NA	NA	Y	Y	Y	Y	Y	Y	Y	Y	Y	81.82
Li et al. [80]	2018	China	NA	NA	Y	Y	Y	Y	Y	Y	Y	Y	Y	81.82
Rema et al. [89]	2018	India	NA	NA	Y	Y	Y	Y	Y	Y	Y	Y	Y	81.82
Romeo et al. [82]	2018	Argentina	NA	NA	Y	Y	Y	Y	Y	Y	Y	Y	Y	81.82
Tortorella et al. [91]	2018	USA	NA	NA	Y	Y	Y	Y	Y	Y	Y	Y	Y	81.82
Bizzarri et al. [83]	2019	Italy	NA	NA	Y	Y	Y	Y	Y	Y	Y	Y	Y	81.82
Gregorio et al. [84]	2019	Germany	NA	NA	Y	Y	Y	Y	Y	Y	Y	Y	Y	81.82
Kiiski et al. [85]	2019	Finland	NA	NA	Y	Y	Y	Y	Y	Y	Y	Y	Y	81.82
Lago et al. [86]	2019	Spain	NA	NA	Y	Y	Y	Y	Y	Y	Y	Y	Y	81.82
Nedyalkov et al. [88]	2019	Bulgaria	NA	NA	Y	Y	Y	Y	Y	Y	Y	Y	Y	81.82
Ichihara et al. [111]	2019	Japan	NA	NA	Y	Y	Y	Y	Y	Y	Y	Y	Y	81.82
Lewandowska et al. [92]	2020	Poland	NA	NA	Y	Y	Y	Y	Y	Y	Y	Y	Y	81.82
Vigneswaran et al. [94]	2020	USA	NA	NA	Y	Y	Y	Y	Y	Y	Y	Y	Y	81.82
Nonaka et al. [112]	2020	Japan	NA	NA	Y	Y	Y	Y	Y	Y	Y	Y	Y	81.82
Bogner et al. [95]	2021	Germany	NA	NA	Y	Y	Y	Y	Y	Y	Y	Y	Y	81.82
Kanao et al. [97]	2021	Japan	NA	NA	Y	Y	Y	Y	Y	Y	Y	Y	Y	81.82
Nielsen et al. [102]	2022	Denmark	NA	NA	Y	Y	Y	Y	Y	Y	Y	Y	Y	81.82
Rios-Doria et al. [113]	2022	USA	NA	NA	Y	Y	Y	Y	Y	Y	Y	Y	Y	81.82
Abdulrahman et al. [99]	2022	UK	NA	NA	Y	Y	Y	Y	Y	Y	Y	Y	Y	81.82
Quyn et al. [115]	2023	UK	NA	NA	Y	Y	Y	Y	Y	Y	Y	Y	Y	81.82
Naha et al. [116]	2023	USA	NA	NA	Y	Y	Y	Y	Y	Y	Y	Y	Y	81.82
Ralston et al. [117]	2023	UK	NA	NA	Y	Y	Y	Y	Y	Y	Y	Y	Y	81.82
Saqib et al. [118]	2023	UK	NA	NA	Y	Y	Y	Y	Y	Y	Y	Y	Y	81.82
Beppu et al. [119]	2023	Japan	NA	NA	Y	Y	Y	Y	Y	Y	Y	Y	Y	81.82
Valstad et al. [120]	2023	Norway	NA	NA	Y	Y	Y	Y	Y	Y	Y	Y	Y	81.82

JBI: Joanna Briggs Institute, NA: not applicable, Y: yes, N: no

Q1: Were the two groups similar and recruited from the same population?

Q2: Were the exposures measured similarly to assign people to both exposed and unexposed groups?

Q3: Was the exposure measured in a valid and reliable way?

Q4: Were confounding factors identified?

Q5: Were strategies to deal with confounding factors stated?

Q6: Were the groups/participants free of the outcome at the start of the study (or at the moment of exposure)?

Q7: Were the outcomes measured in a valid and reliable way?

Q8: Was the follow up time reported and sufficient to be long enough for outcomes to occur?

Q9: Was follow up complete, and if not, were the reasons to loss to follow up described and explored?

Q10: Were strategies to address incomplete follow up utilized?

Q11: Was appropriate statistical analysis used?

**Table 3:** Quality assessment of case series according to JBI checklist

Author	Publish Year	Country	Q1	Q2	Q3	Q4	Q5	Q6	Q7	Q8	Q9	Q10	Quality Score (%)
Thornton et al. [18]	1973	USA	yes	yes	yes	yes	yes	yes	yes	yes	yes	yes	100
Eckhauser et al. [20]	1979	USA	yes	yes	yes	yes	yes	yes	yes	yes	yes	yes	100
Takagi et al. [24]	1985	Japan	yes	yes	yes	yes	yes	yes	yes	yes	yes	yes	100
Sardi et al. [29]	1994	USA	yes	yes	yes	yes	yes	unclear	yes	yes	yes	yes	90
Woodhouse et al. [30]	1995	UK	yes	yes	yes	unclear	yes	unclear	yes	yes	yes	yes	80
Shirouzu et al. [32]	1996	Japan	yes	yes	yes	yes	yes	yes	yes	yes	yes	yes	100
Kamat et al. [13]	2003	USA	yes	yes	yes	yes	yes	yes	yes	yes	yes	yes	100
Leibovici et al. [42]	2005	USA	yes	yes	yes	yes	yes	yes	yes	yes	yes	yes	100
Ferron et al. [107]	2006	France	yes	yes	yes	yes	yes	yes	yes	yes	yes	yes	100
Puntambekar et al. [51]	2009	India	yes	yes	yes	yes	yes	unclear	yes	yes	yes	yes	90
Guimarães et al. [56]	2011	Brazil	yes	yes	yes	yes	yes	yes	yes	yes	yes	yes	100
Tanaka et al. [68]	2014	Japan	yes	yes	yes	yes	yes	yes	yes	yes	yes	yes	100
Xin et al. [69]	2014	Singapore	yes	yes	yes	yes	yes	yes	yes	yes	yes	yes	100
Căpîlna et al. [70]	2015	Romania	yes	yes	yes	yes	yes	unclear	yes	yes	yes	yes	90
Moreno-Palacios et al. [73]	2015	Spain	yes	yes	yes	yes	yes	yes	yes	yes	yes	yes	100
Yang et al. [109]	2015	China	yes	yes	yes	yes	yes	yes	yes	yes	yes	yes	100
Mehta et al. [81]	2018	UK	yes	yes	yes	unclear	yes	yes	yes	yes	yes	yes	90
Pokharkar et al. [110]	2018	India	yes	yes	yes	yes	yes	yes	yes	unclear	yes	yes	90
Lee et al. [87]	2019	Australia	yes	yes	yes	yes	yes	unclear	yes	yes	yes	yes	90
Soeda et al. [90]	2019	Japan	yes	yes	yes	yes	yes	unclear	yes	yes	yes	yes	100
Tuech et al. [93]	2020	France	yes	yes	yes	yes	yes	yes	yes	yes	yes	yes	100
Brown et al. [96]	2021	Australia	yes	yes	yes	yes	yes	yes	yes	yes	yes	yes	100
Karkia et al. [114]	2022	UK	yes	yes	yes	yes	yes	yes	yes	yes	yes	yes	100

JBI: Joanna Briggs Institute, U: unclear, Y: yes, N: no

Q1: Were there clear criteria for inclusion in the case series?

Q2: Was the condition measured in a standard, reliable way for all participants included in the case series?

Q3: Were valid methods used for identification of the condition for all participants included in the case series?

Q4: Did the case series have consecutive inclusion of participants?

Q5: Did the case series have complete inclusion of participants?

Q6: Was there clear reporting of the demographics of the participants in the study?

Q7: Was there clear reporting of clinical information of the participants?

Q8: Were the outcomes or follow up results of cases clearly reported?

Q9: Was there clear reporting of the presenting site(s)/clinic(s) demographic information?

Q10: Was statistical analysis appropriate?

### Quantitative data synthesis and heterogeneity across studies

#### Colorectal cancers mortality rate

The MR following TPE in colorectal cancers was estimated and the results of meta-analysis suggested that intra-operative MR is 0.2% (*n* = 27, 95%CI = 0.07–1.11%, I-square = 0.0%), in-hospital MR is 3.11% (*n* = 31, 95%CI = 2.15–4.46%, I-square = 9.02%), 30-day MR is estimated as 2.61% (*n* = 35, 95%CI = 1.95–3.48%, I-square = 15.18%), 90-day MR is 6.22% (*n* = 12, 95%CI = 4.17–9.18%, I-square = 16.87%) and overall MR is estimated as 30.57% (*n* = 13, 95%CI = 26.9–34.4%, I-square = 60.6%), respectively (Table 4). All analysis was done by fixed-effects model because of no significant heterogeneity among studies. In addition, the overall MR in open surgery was 30.57%, in primary cancer 2.44%, and in primary and recurrent cancers 31.6%. There were not enough studies to perform meta-analysis for recurrent cancer.

**Table 4:** Summary of meta-analysis to estimate the mortality rate following TPE in colorectal cancers

Subgroups	Time	Number of included studies	Fixed effect models		Random effect models		I square
			Mortality rate	95%CI	Mortality rate	95%CI	
Overall	Intra-operative mortality	27	0.28%	0.07–1.11%	0.28%	0.07–1.11%	0.0%
	In-hospital Mortality	31	3.11%	2.15–4.46%	1.44%	0.52–3.93%	9.02%
	30-day Mortality	35	2.61%	1.95–3.48%	2.30%	1.17–4.49%	15.18%
	90-day Mortality	12	6.22%	4.17–9.18%	2.96%	0.82–10.1%	16.87%
	Overall-mortality	13	30.57%	26.9–34.4%	31.88%	23.8-41.26%	60.6%
Open surgery	Intra-operative mortality	25	0.29%	0.07–1.16%	0.29%	0.07–1.16%	0.0%
	In-hospital Mortality	29	3.23%	2.24–4.63%	1.59%	0.59–4.20%	10.15%
	30-day Mortality	34	2.64%	1.97–3.53%	2.42%	1.25–4.64%	16.48
	90-day Mortality	12	6.39%	4.28–9.43%	3.24%	0.94–10.5%	17.66%
	Overall-mortality	13	30.57%	26.9–34.4%	31.88%	23.8-41.26%	60.6%
Minimally invasive surgery	Intra-operative mortality	4	0%	0.00-100%	-	-	0.0%
	In-hospital Mortality	4	0%	0.00-100%	-	-	0.0%
	30-day Mortality	3	0%	0.00-100%	-	-	0.0%
	90-day Mortality	Insufficient data to perform meta-analysis					
	Overall-mortality	Insufficient data to perform meta-analysis					
Primary and Recurrent	Intra-operative mortality	16	0.0	0-11.47%	-	-	0.0%
	In-hospital Mortality	19	3.16%	2.0-4.96%	1.03%	0.21–4.97%	9.23%
	30-day Mortality	25	2.59%	1.88–3.56%	2.08%	0.84–5.04%	17.5%
	90-day Mortality	8	6.95%	4.6-10.43%	2.44%	0.33–15.76%	2.13%
	Overall-mortality	8	31.6%	27.5–36.2%	34.89%	26.85–43.9%	57.2%
Primary	Intra-operative mortality	8	0.91%	0.23–3.56%	0.91%	0.23–3.56%	0.0%
	In-hospital Mortality	9	4.50%	2.4–8.17%	4.22%	1.82–9.47%	2.99%
	30-day Mortality	7	4.64%	2.23–9.40%	4.64%	2.23–9.40%	0.0%
	90-day Mortality	2	3.33%	0.84–12.3%	3.33%	0.84–12.37%	0.0%
	Overall-mortality	4	27.6%	20.71–35.8%	2.44%	8.9–51.5%	72.0%
Recurrent	Intra-operative mortality	5	0%	0.00-100%	-	-	0.0%
	In-hospital Mortality	5	0%	0.00-100%	-	-	0.0%
	30-day Mortality	5	0%	0.00-100%	-	-	0.0%
	90-day Mortality	2	0%	0.00-100%	0%	0.00-100%	0.0%
	Overall-mortality	Insufficient data to perform meta-analysis					

### Gynecological cancers mortality rate

Regarding MR following TPE in gynecological cancers, the obtained results showed that intra-operative MR is 0.21% (*n* = 40, 95%CI = 0.05–0.85%, I-square = 0.0%), in-hospital MR is 2.65% (*n* = 34, 95%CI = 1.61–4.36%, I-square = 1.35%), 30-day MR is estimated as 5.89% (*n* = 37, 95%CI = 4.65–7.43%, I-square = 0.39%), 90-day MR is 2.74% (*n* = 7, 95%CI = 1.03–7.07%, I-square = 0.0%) and overall MR is estimated as 25.5% (*n* = 12, 95%CI = 19.8–32.1%, I-square = 46.6%), respectively (Table 5). All analysis was done by fixed-effects model because of no significant heterogeneity among studies. The overall MR in open surgery was 25.5%, in minimally invasive surgery was 25.0%, and in primary, recurrent and both of them together was 53.8%, 12.7% and 55.5%, respectively.

**Table 5:** Summary of meta-analysis to estimate the mortality rate following TPE in gynecological cancers

Subgroups	Time	Number of included studies	Fixed effect models		Random effect models		I square
			Mortality rate	95%CI	Mortality rate	95%CI	
Overall	Intra-operative mortality	40	0.21%	0.05–0.85%	0	0–0	0%
	In-hospital Mortality	34	2.65%	1.61–4.36%	0.51%	0.07–3.72%	1.35%
	30-day Mortality	37	5.89%	4.65–7.43%	0.32%	0.04–2.70%	0.39%
	90-day Mortality	7	2.74%	1.03–7.07%	2.74%	1.03–7.07%	0.0%
	Overall-mortality	12	25.5%	19.8–32.1%	35.29%	15.3–62.1%	46.6%
Open surgery	Intra-operative mortality	35	0.22%	0.05–0.87%	0	0–0	0%
	In-hospital Mortality	29	2.79%	1.69–4.58%	0.59%	0.08–4.07%	2.33%
	30-day Mortality	32	6.04%	4.77–7.61%	0.38%	0.05–2.99%	0.70%
	90-day Mortality	3	3.08%	1.16–7.91%	3.08%	1.16–7.91%	0.0%
	Overall-mortality	8	25.5%	19.7–32.5%	44.82%	19.4–73.2%	67.5%
Minimally invasive surgery	Intra-operative mortality	5	0%	0.00-100%	-	-	0.0%
	In-hospital Mortality	5	0%	0.00-100%	-	-	0.0%
	30-day Mortality	5	0%	0.00-100%	-	-	0.0%
	90-day Mortality	4	0%	0.00-100%	-	-	0.0%
	Overall-mortality	4	25.0	9.71–50.8%	5.51%	0.01–96.5%	2.68%
Primary	Intra-operative mortality	4	0%	0.00-100%	-	-	0.0%
	In-hospital Mortality	7	3.85%	0.96–14.1%	3.85%	0.96–14.1%	0.0%
	30-day Mortality	6	2.04%	0.29–13.1%	1.89%	0.06–39.8%	1.17%
	90-day Mortality	2	0%	0.00-100%	0%	0.00-100%	0.0%
	Overall-mortality	3	53.8%	28.1–77.6%	53.8%	28.1–77.6%	0.0%
Recurrent	Intra-operative mortality	10	0.87%	0.19–3.05%	0%	0.00-100%	0.0%
	In-hospital Mortality	8	1.54%	0.50–4.66%	0.99%	0.09–9.52%	3.23%
	30-day Mortality	9	1.52%	0.49–4.61%	0.97%	0.09–9.36%	2.81%
	90-day Mortality	4	1.69%	0.42–6.5%	1.69%	0.42–6.5%	0.0%
	Overall-mortality	6	12.7%	8.03–19.4%	18.6%	2.63–65.8%	23.7%
Primary and Recurrent	Intra-operative mortality	26	0%	0.00-100%	-	-	0.0%
	In-hospital Mortality	19	3.14%	1.70–5.74%	0.01%	0-98.02%	0.0%
	30-day Mortality	23	7.1%	5.56–8.99%	0.14%	0-5.42%	0.08%
	90-day Mortality	Insufficient data to perform meta-analysis					
	Overall-mortality	3	55.5%	40.9–69.2%	55.5%	40.9–69.2%	0.0%

### Urological cancers mortality rate

In the case of urological cancers, there have been fewer studies, but still, the results showed that 30-day MR is estimated as 2.07% (*n* = 4, 95%CI = 1.37–3.13%, I-square = 0.0%).

### Miscellaneous cancers mortality rate

The results of meta-analysis revealed that following TPE in Miscellaneous cancers, MR of intra-operative MR is 0.16% (*n* = 16, 95%CI = 0.02–1.1%, I-square = 56.9%), in-hospital MR is 0.8% (*n* = 17, 95%CI = 0.3–2.12%, I-square = 57.6%), 30-day MR is estimated as 1.59% (*n* = 18, 95%CI = 1.23–2.04%, I-square = 6.01%), 90-day MR is 2.33% (*n* = 4, 95%CI = 1.11–4.8%, I-square = 0.0%), and overall MR is estimated as 12.42% (*n* = 3, 95%CI = 9.2–16.6%, I-square = 39.7%) (Table 6). These rates for surgeries are reported in Table 6, but for other cases, due to the lack of sufficient studies, meta-analysis was not performed.

**Table 6:** Summary of meta-analysis to estimate the mortality rate following TPE in Misc. cancers

Subgroups	Time	Number of included studies	Fixed effects model		Random effects model		I square
			Mortality rate	95%CI	Mortality rate	95%CI	
Overall	Intra-operative mortality	16	0.16%	0.02–1.10%	0.06%	0-15.87%	56.9%
	In-hospital Mortality	17	0.80%	0.30–2.12%	0.78%	0.17–3.46%	57.6%
	30-day Mortality	18	1.59%	1.23–2.04%	0.53%	0.09–3.18%	6.01%
	90-day Mortality	4	2.33%	1.11–4.80%	2.33%	1.11–4.80	0.0%
	Overall-mortality	3	12.42%	9.2–16.6%	9.90%	4.37–20.9%	39.7%
Open surgery	Intra-operative mortality	13	0.17%	0.02–1.17%	0.06%	0-16.5%	76.9%
	In-hospital Mortality	14	0.9%	0.3–2.3%	0.86%	0.20–3.59%	23.5%
	30-day Mortality	15	1.60%	1.25–2.07%	0.60%	0.11–3.33%	9.44%
	90-day Mortality	3	2.41%	1.16–4.98%	2.41%	1.16–4.98%	0.0%
	Overall-mortality	2	12.2%	8.9–16.4%	12.2%	8.9–16.4%	-

## Discussion

In this study, we investigated the MR after TPE using meta-analysis method, which included different types of cancers such as colorectal, gynecological, urological and miscellaneous cancers. The main findings of this study showed that the highest mortality rate was related to overall mortality. The overall mortality rate was 30.57% in colorectal cancers, 25.5% in gynecological cancers and 12.42% in Miscellaneous. In fact, the highest rate of mortality is related to the overall mortality rate of colorectal cancers. Naturally, the MR in open surgeries was higher than in minimally invasive surgeries, and also in primary advanced cancers, it was higher than in recurrent cancers.

Generally, TPE is used in the treatment of advanced gynecological cancers as well as primary advanced and recurrent rectal cancers, so it is mostly used in cases where conventional treatment modalities do not have a suitable prognosis. Due to the fact that the stage of cancer is higher and the prognosis is worse in patients who are candidates for this surgery, it is expected that the MR will be higher, on the other hand, this surgery is considered as an advanced surgery, and its success rate depends on the experience of the surgeon and the equipment of the surgical center.

In a study by Vigneswaran et al. [94] with the largest sample size conducted in the USA, 2305 cases of TPE between 2005 and 2016 were evaluated. Of these, 45% were urological malignancies, 33% colorectal, 15% gynecological and 9% other cancers. The authors have stated that despite the common complications in this surgery, the mortality rate is relatively low and the outcomes during and after the operation are dissimilar in different types of cancer. Also, the prevalence of major complications is 15%, 30-day mortality is 2%, the duration of hospitalization after surgery is 9 days, and blood transfusion is reported in 50% of cases. The results of the present meta-analysis estimated the 30-day mortality rate to be 2.61%, 5.89%, 1.59% and 2.07% in colorectal, gynecological, miscellaneous and urological cancer which is higher than the value reported in the aforementioned study in most cases. Part of this difference can be related to better equipment and care in USA medical centers and part of it to more experience of medical centers and surgical teams. In our study, the results showed that the overall mortality rate in gynecological malignancies is lower than that in colorectal cancers (25.5% vs. 30.57%). Although in the study of Vigneswaran et al. [94], no significant difference was reported in the 30-day mortality rate of different cancers, but the prevalence of complications was higher in gynecological cancers, and the return to the operating room due to complications was also higher in gynecological cancers than in colorectal cancer (12.8% vs. 8.7%), while it was 4.8% for urological cancers.

Intra-operative mortality rate in colorectal cancers with rate of 0.21% showed the highest rate among studied cancers and its value in all other cancers were 0.2% or less. In terms of in-hospital mortality, this rate was estimated at 3.11% for colorectal cancer, and the highest rate of in-hospital mortality rate was related to colorectal cancer. Therefore, the results of our study showed that in performing TPE for colorectal cancers, intraoperative, in-hospital, 30-day, 90-day and overall mortality rate is more than gynecological, urological, and miscellaneous cancers.

It is important to note that while recent advancements in surgical techniques and well-equipped surgical centers have improved mortality rates for TPE, the main rationale for such an aggressive surgery is the potential chance for a cure, which has been reported in up to 63% of patients [103]. However, the effectiveness of alternative options such as robotic-assisted or laparoscopic surgeries in achieving this goal has not been thoroughly studied [104]. One notable study by Bizzarri et al. [83] reported a 30-day mortality rate of 0% following minimally invasive TPE, demonstrating its feasibility in a small group of 5 patients. More research is needed to fully understand the outcomes of minimally invasive TPE compared to conventional surgical method.

The complexity of the TPE procedure makes it challenging to predict outcomes. Factors such as the purpose of surgery (curative or palliative), cancer type, patient comorbidities, and the expertise of the surgical team and center are known to be associated with morbidity and mortality [94, 104, 105]. Patients undergoing TPE also require strong physical and emotional support. Therefore, a skilled multi-disciplinary team is essential for evaluating patient eligibility and performing the surgery. Previous studies have emphasized the use of specific guidelines, such as the enhanced recovery after surgery (ERAS) guideline, to reduce complications [94, 106]. Ultimately, individualized patient selection is recommended before performing TPE.

To the best of our knowledge, this is the largest meta-analysis of MR following TPE. However, several limitations should be acknowledged. Our data may be biased towards reporting more studies with a 0% MR. This is mainly because if a study reported a 0% MR for a specific time period, the MR for previous periods would be assumed to be 0% as well, even if it wasn’t reported in detail. However, if a study reported a MR higher than 0% for a specific time period and didn’t report the previous MRs, those data points were labeled as missing. Furthermore, in this study we included as much studies as possible, to create a holistic picture of MR following TPE. Therefore, it might be subject to bias as all TPE performed since 1976 with proper definition of TPE were included in our analysis. Further studies are required to investigate the impact of surgical intention, surgical center expertise, post-operation care, and patients’ comorbidities on MR following TPE.

## Conclusion

In conclusion, it can be said that performing TPE in a specialized surgical center with careful patient eligibility evaluation is a viable option for advanced malignancies of the pelvic organs.

## Data Availability

All data generated or analyzed during this study are included in the article.
